# Giant cell arteritis: A population-based retrospective cohort study exploring incidence and clinical presentation in Canterbury, Aotearoa New Zealand

**DOI:** 10.3389/fmed.2022.1057917

**Published:** 2022-11-22

**Authors:** Suellen A. Lyne, Carlee Ruediger, Susan Lester, Peter T. Chapman, Ernst Michael Shanahan, Catherine L. Hill, Lisa Stamp

**Affiliations:** ^1^School of Medicine, University of Adelaide, Adelaide, SA, Australia; ^2^Department of Rheumatology, The Queen Elizabeth Hospital, Adelaide, SA, Australia; ^3^Department of Rheumatology, Flinders Medical Centre, Adelaide, SA, Australia; ^4^Department of Rheumatology, Te Whatu Ora Waitematā, Christchurch, New Zealand; ^5^College of Medicine and Public Health, Flinders University, Adelaide, SA, Australia; ^6^Department of Rheumatology, Royal Adelaide Hospital, Adelaide, SA, Australia; ^7^School of Medicine, University of Otago, Christchurch, New Zealand

**Keywords:** epidemiology, giant cell arteritis, incidence, vasculitis, large vessel vasculitis (LVV)

## Abstract

**Background/aim:**

To determine the epidemiology and clinical features of giant cell arteritis (GCA) in Canterbury, Aotearoa New Zealand, with a particular focus on extra-cranial large vessel disease.

**Methods:**

Patients with GCA were identified from radiology and pathology reports, outpatient letters and inpatient hospital admissions in the Canterbury New Zealand from 1 June 2011 to 31 May 2016. Data was collected retrospectively based on review of electronic medical records.

**Results:**

There were 142 cases of GCA identified. 65.5% of cases were female with a mean age of 74.2 years. The estimated population incidence for biopsy-proven GCA was 10.5 per 100,000 people over the age of 50 and incidence peaked between 80 and 84 years of age. 10/142 (7%) people were diagnosed with large vessel GCA, often presenting with non-specific symptoms and evidence of vascular insufficiency including limb claudication, vascular bruits, blood pressure and pulse discrepancy, or cerebrovascular accident. Those with limited cranial GCA were more likely to present with the cardinal clinical features of headache and jaw claudication. Patients across the two groups were treated similarly, but those with large vessel disease had greater long-term steroid burden. Rates of aortic complication were low across both groups, although available follow-up data was limited.

**Conclusion:**

This study is the first of its kind to describe the clinical characteristics of large vessel GCA in a New Zealand cohort. Despite small case numbers, two distinct subsets of disease were recognized, differentiating patients with cranial and large vessel disease. Our results suggest that utilization of an alternative diagnostic and therapeutic approach may be needed to manage patients with large vessel disease.

## Introduction

Giant Cell Arteritis (GCA) is the most common vasculitis affecting people over the age of 50 years. Highest rates are observed in people with Scandinavian ancestry and epidemiological characteristics have been well-described in large populations across Europe and Northern America (1, 2). Little work has been published on the epidemiology of GCA in Aotearoa New Zealand (NZ). One retrospective cohort study reported a mean annual incidence of 12.7 per 100,000 over the age of 50 for biopsy-proven GCA (3) and a recent study assessing diagnostic performance of color duplex ultrasound reported an incidence in NZ Europeans and Māori of 13.2 and 12.2, respectively (4). Additional work has been conducted exploring seasonal influence on rates of GCA in NZ, with no meaningful trends identified (5).

GCA is a clinically heterogenous disease characterized by granulomatous inflammation of medium and large vessels. Traditionally described as a disease of the temporal arteries, it is now understood to be a systemic disease involving the aorta and any of its major tributaries (6–8). Three primary disease subtypes are recognized: classical or “pure” cranial GCA (C-GCA); extracranial manifestations in the context of established cranial disease; or isolated extracranial large vessel disease without cranial manifestations. The latter two are both designated large vessel GCA (LV-GCA). Each of these phenotypes may occur with or without co-existent symptoms of polymyalgia rheumatica (PMR) (9).

The extent and distribution of vascular involvement in LV-GCA can vary considerably and presenting symptoms are often non-specific. Diagnosis may be difficult as LV-GCA patients are less likely to yield a positive temporal artery biopsy (TAB) and less likely to meet the 1990 American College of Rheumatology (ACR) classification criteria for a diagnosis of GCA, which rely heavily on cranial manifestations (10, 11). Patients with GCA are 17 times more likely to develop thoracic aortic aneurysm compared to age and sex-matched controls and occurrence of this complication is associated with increased mortality (12, 13). Detection of LV involvement is crucial because complications are potentially catastrophic and may not present until years after diagnosis (14–16).

Despite increased awareness of LV involvement in GCA and its potential complications, there is still a paucity of knowledge regarding true incidence rates, implications on treatment strategies and surveillance of long term sequelae. To our knowledge, characteristics of extracranial manifestations, irrespective of cranial involvement, have never been described in a NZ cohort. This proposed research seeks to further our understanding of the epidemiology, clinical manifestations, and complications GCA in NZ, with a particular focus on extra-cranial disease, thereby guiding future screening and management protocols.

## Materials and methods

### Study design

This retrospective cohort study included incident cases of GCA diagnosed in the Te Whatu Ora Waitaha Canterbury (formerly Canterbury District Health Board) between 1 June 2011 and 31 May 2016. This study was developed in consultation with Māori researcher groups and was approved by the University of Otago, Human Research Ethics Committee (Reference: H21/065).

### Inclusion and exclusion criteria

All male and female patients with incident GCA were included. Fulfillment of the 1990 American College of Rheumatology (ACR) classification criteria (10) was not required, with the exception of age > 50, as these criteria are known to preclude patients with isolated extra-cranial disease (11). A positive temporal artery biopsy (TAB) was not required; however, a diagnosis of biopsy-negative GCA had to be confirmed by the treating Rheumatologist, Ophthalmologist, Neurologist, or General Physician. Patients were excluded if an alternative cause for large vessel vasculitis (LVV), such as Takayasu, was confirmed or suspected.

### Case identification

Case identification was based on keyword search of radiology reports, histopathology reports and rheumatology outpatient letters, as well as International Coding of Disease (ICD) classification for inpatient admissions. Keywords included “*temporal arteritis*,” *“giant cell arteritis*,” “*arteritis*,” “*aortitis*,” “*vasculitis*,” *and* “*Takayasu.*” Radiology reports were derived from all imaging modalities at Christchurch Hospital as well as private radiology providers within Canterbury. Features compatible with a diagnosis of vasculitis included circumferential wall thickening, with or without contrast enhancement, and/or vascular stenosis/occlusion, and/or vascular dilation/aneurysm. It was the final opinion of the radiologist that determined the assignment of a positive or negative study. All histopathology reports from Canterbury Health Laboratories were reviewed, except skin and renal specimens, which limited results returning with small vessel vasculitis. Once cases were identified, available electronic medical records were reviewed to confirm the diagnosis of GCA.

### Data collection

Data collection included demographics, time to diagnosis, presenting clinical features, biopsy and laboratory results, distribution of large vessel involvement, disease complications, treatment, and treatment related outcomes. Duration of follow-up was determined by the last clinical encounter, up until 31 May 2021, allowing a minimum 5-year follow-up for all patients. Refer to Supplementary File 1 for the data extraction table.

### Study definitions

For the purposes of the study, patients were classified into two groups: those with limited cranial GCA (C-GCA) and those with extra-cranial large vessel GCA (LV-GCA). The latter was defined by the presence of extra-cranial vasculitis on imaging or histopathology, as designated by the reporting radiologist or pathologist. Clinical features suggesting large vessel disease included upper or lower limb claudication, vascular bruits, blood pressure or peripheral pulse discrepancy, aortic aneurysm, dissection or rupture, evidence of ischemic sequelae or end organ infarction due to large vessel stenosis (17).

### Statistical analysis

Descriptive data are presented as frequencies and percentages for categorical variables and mean with standard deviation (SD) for continuous variables. The frequency of a specific feature is stated as the number of cases with that feature/number of cases in which the feature was detailed, except in the case of clinical symptoms, where symptoms that were not reported were assumed to be absent. Univariate analysis using Fisher’s Exact test was used to study categorical variables. *T*-tests were used to compare the mean age at diagnosis. Mean incidence rate was estimated by Poisson regression, based on 2013 census data available through *Tatauranga Aotearoa*.^1^ Stata software (17.0, StataCorp LLC, College Station, TX) was used for data synthesis. All significance tests were two-tailed and values of *p* < 0.05 were considered significant.

## Results

**FIGURE 1:**
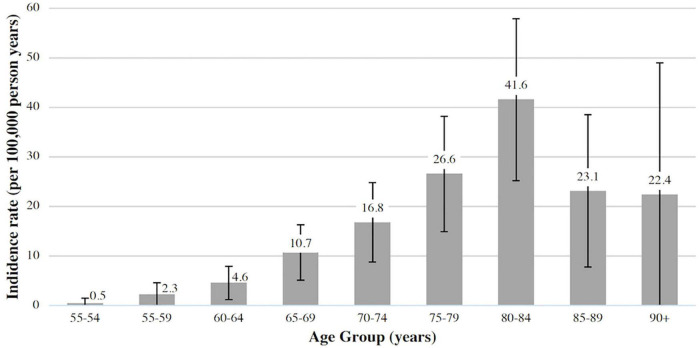
Incidence of biopsy-proven giant cell arteritis (GCA) in Canterbury (adjusted for age and sex, 95% CI).

One hundred forty-two patients with incident GCA were identified in the study period. 100 (70.4%) were biopsy-proven GCA; of the remaining 42 cases, 7 did not undergo a biopsy, 2 were technically unsuccessful and 33 had negative biopsies. The mean annual incidence for biopsy-proven GCA (*n* = 100) was 10.5 (95% CI 8.7, 12.8) per 100,000 over the age of 50, and this increased to 15 (95% CI 12.6, 17.6) when all cases were included (*n* = 142). Patients were predominantly female (65.5%), with a mean ± SD age at diagnosis of 74.2 ± 8.9 years. Incidence rates were highest after 65 years of age and peaked between 80 and 84 years (Figure 1).

10/142 (7%) had LV-GCA confirmed on imaging and 7 of the remaining 132 patients with limited C-GCA had symptoms suggestive of possible extra-cranial involvement, but without confirmation on imaging or histopathology. Baseline characteristics of the LV-GCA and C-GCA cohorts are summarized in Table 1. There was no statistically significant difference in baseline demographics between the two groups. The mean delay from symptom onset to diagnosis was longer in the LV-GCA group at 11.6 weeks, compared to 6.7 weeks, although statistical significance was not met (*p* = 0.08). Patients with C-GCA were more likely to present with headache and jaw claudication, while those with LV-GCA were more likely to experience weight loss, upper limb claudication, vascular bruits, blood pressure and pulse discrepancy or cerebrovascular accident. There was no difference in baseline laboratory parameters including CRP, ESR, platelet count and hemoglobin. Those with C-GCA were more likely to undergo a TAB (*p* = 0.008), but there was no difference in the rate of biopsy positivity between the two groups (*p* = 0.38). Only 50% of the patients with LV-GCA fulfilled the 1990 ACR classification criteria, which was significantly less than the 82% seen the C-GCA group (*p* = 0.03).

**TABLE 1:** Baseline characteristic of patients with limited cranial giant cell arteritis (GCA) compared to those with large vessel GCA.

	Large Vessel GCA (*n* = 10)		Cranial GCA (*n* = 132)		*P*-value (<0.05)
**Demographics**					
Gender (female)	6/10	60%	87/132	65.9%	0.74
Age at diagnosis (mean ± SD)	70.6 ± 9.6		74.5 ± 8.8		0.19
New Zealand European	8/9	88.9%	114/122	93.4%	0.48
Māori	1/9	11.1%	0/122	0%	0.08
Pacifica	0/9	0%	3/122	2.6%	0.81
**Presenting clinical features (n/N, %)**					
Delay to diagnosis in weeks (mean ± SD)	11.6 ± 8.8		6.7 ± 7.5		0.08
Cranial manifestations	3/10	30%	115/124	92.7%	**< 0.001**
Headache	3/10	30%	103/123	83.7%	**0.001**
Jaw claudication	1/10	10%	57/122	46.7%	**0.042**
Scalp tenderness	3/10	30%	70/122	57.4%	0.11
Transient visual disturbance	3/10	30%	41/123	33.3%	1.00
Permanent vision loss	2/10	20%	18/123	14.6%	0.65
Systemic/constitutional manifestations	9/10	90%	89/124	71.8%	0.29
Polymyalgia rheumatica	5/10	50%	68/124	54.8%	1.00
Fever	3/10	30%	35/123	28.5%	1.00
Weight loss	6/10	60%	28/123	22.8%	**0.018**
Cough	3/10	30%	10/123	8.1%	0.059
Features of extra-cranial disease	10/10	100%	7/123	5.7%	**< 0.001**
Upper limb claudication	3/10	30%	1/123	0.8%	**0.001**
Lower limb claudication	1/10	10%	2/123	1.6%	0.21
Vascular bruits	3/10	30%	0/123	0%	**< 0.001**
Blood pressure discrepancy	2/10	20%	1/123	0.8%	**0.015**
Pulse discrepancy	5/10	50%	0/123	0%	**< 0.001**
Aortic aneurysm at diagnosis	2/10	20%	1/123	0.8%	**0.015**
Cerebrovascular accident	3/10	30%	2/123	1.6%	**0.003**
**Laboratory tests (mean ± SD)**					
CRP (mg/L)	95.4 ± 79.1		91.5 ± 88.5		0.89
ESR (mm/h)	66.3 ± 30.2		53.4 ± 30.8		0.26
Platelets (×10^9^/L)	413.9 ± 152		384.4 ± 158.7		0.57
Hemoglobin (g/L)	112.1 ± 15.7		125.8 ± 24.5		0.08
**Temporal artery biopsy**					
TAB Performed	7/10	70%	128/132	97%	**0.008**
TAB Positive	4/7	57%	96/128	75%	0.38
**1990 ACR criteria**					
Fulfilled at least 3/5 ACR criteria	5/5	50%	100/122	82%	**0.030**

The bold numbers are those *P* values that meet statistical significance (< 0.05).

**FIGURE 2:**
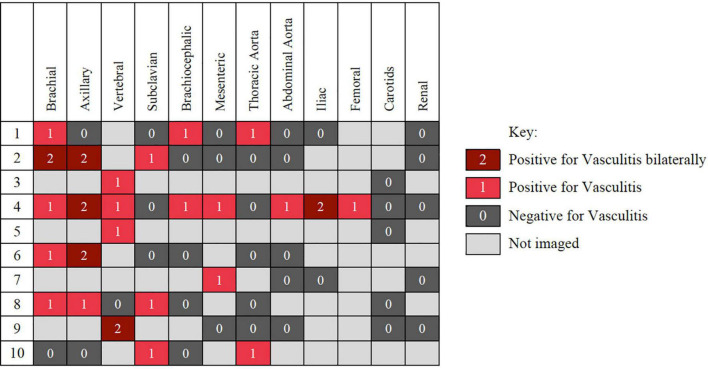
Distribution of disease involvement in the 10 patient with large vessels vasculitis detected on computed tomography angiography (CT-A), magnetic resonance angiography (MR-A), and ultrasound (US).

Imaging modalities used to detect LV-GCA were computed tomography angiography (CT-A), magnetic resonance angiography (MR-A), and ultrasound (US), with 7, 4, and 1 studies, respectively, positive. Imaging was conducted either to evaluate symptoms of vascular disease, such as stroke or limb claudication, or as work up for pyrexia of unknown origin (PUO) (*n* = 2). Arterial involvement was most frequently detected in the proximal branches of the aorta, with brachial, axillary and vertebral most frequently involved. Of those with imaging of the brachial arteries 83% were positive, 66.7% for axillary arteries and 80% for vertebral arteries (Figure 2).

Patients with LV- and C-GCA were universally managed with corticosteroid therapy, with no difference in starting dose of oral prednisolone. There was also no difference in the number of patients requiring intravenous (IV) methylprednisolone, although indications differed. Of the two LV-GCA patients requiring IV methylprednisolone, one was for management of arm ischemia and the other was for a posterior circulation stroke with co-existent vision impairment secondary to central retinal artery occlusion (CRAO). IV Methylprednisolone in the C-GCA group was exclusively used for visual disturbance.

Follow-up data was limited, as information was not available for patients discharged to the care of their General Practitioner (GP). Available data suggests those with LV-GCA were more likely to continue prednisolone 5 years after diagnosis compared to C-GCA patients (*p* = 0.007) (Table 2). Of those continuing prednisolone, there was no difference in mean dose between the two groups at 1-, 3-, and 5-years of follow-up. Use of an alternative immunosuppression was typically reserved for patients with refractory PMR symptoms. Methotrexate was the treatment of choice, with two C-GCA patients also receiving leflunomide, noting Tocilizumab was not available for treatment of GCA during the study period.

**TABLE 2:** Comparison of treatment variables in patients with large vessel giant cell arteritis (GCA) and cranial GCA.

	Large Vessel GCA (*n* = 10)		Cranial GCA (*n* = 132)		*P*-value (<0.05)
**Treatment**					
Starting Prednisolone dose mg (mean ± sd)	52 ± 14.0		59.8 ± 11.8		**0.049**
Need for IV Methylprednisolone at presentation	2/10	20%	14/129	10.9%	0.38
**Continuing Prednisolone (n/N, %)**					
After 1 year	9/9	100%	87/97	89.7%	0.60
After 3 years	7/9	77.8%	47/86	54.7%	0.29
After 5 years	6/7	85.7%	30/95	31.6%	**0.007**
**Mean dose (mg)**					
After 1 year	8.8 ± 4.6		10.6 ± 8.2		0.53
After 3 years	8.3 ± 6.3		6.4 ± 5.2		0.45
After 5 years	8.8 ± 6.3		8.7 ± 11.7		0.98
Patients starting an alternative immunosuppressive agent during follow-up (n/N)	2/10	20%	16/130	12.3%	0.62
Methotrexate	2/2	100%	16/16	100%	–
Leflunomide	0/2	0%	2/16	12.5%	–

The bold numbers are those *P* values that meet statistical significance (< 0.05).

The mean duration of follow-up was 48.3 months for LV-GCA patients (*n* = 9) and 31.6 months for C-GCA patients (*n* = 72) (*p* = 0.1), of those followed up within the public hospital outpatient setting (Table 3). Irreversible vision loss was seen at similar rates across the two groups, but permanent neurological deficit due to stroke seen at higher rates in the LV-GCA group (*p* = 0.001). 5 (3.8%) of the C-GCA patients had a known abdominal aortic aneurysm (AAA) and one (0.8%) had a thoracic aortic aneurysm that pre-dated their GCA diagnosis, two of whom required repair during follow-up. Two C-GCA patients develop a new AAA during follow-up, one requiring surgical repair. Two LV-GCA patients were diagnosed thoracic aortic dilation either at diagnosis or during follow-up, not meeting criteria for aneurysm. This rate was significantly higher than that observed in C-GCA patients (*p* = 0.004). One C-GCA patient developed a Type B thoracic and abdominal aortic dissection, managed conservatively. No dissections were observed in the LV-GCA group.

**TABLE 3:** Comparison of disease outcomes in patients with large vessel GCA (LV-GCA) and cranial GCA (C-GCA).

	Large vessel GCA (*n* = 10)		Cranial GCA (*n* = 132)		*P*-value (<0.05)
**Outcomes**					
Follow-up data available (n/N)	9/10		72/132		–
Mean Duration of Follow-up (months)	48.3 ± 30		31.6 ± 28		0.1
**Disease complications at diagnosis**					
Irreversible Vision Loss (n/N,%)	2/10	20%	12/129	9.3%	0.27
Neurological Deficit Due to stroke (n/N,%)	3/10	30%	1/129	0.8%	**0.001**
**Aortic complications during follow-up**					
Abdominal aortic aneurysm	0/10	0%	2/132	1.5%	1.0
Thoracic aortic dilation	2/10	20%	0/132	0%	**0.004**
Aortic dissection or rupture	0/10	0%	1/132	0.8%	1.0

The bold numbers are those *P* values that meet statistical significance (< 0.05).

## Discussion

To our knowledge this is the only study to describe clinical characteristics of patients with LV-GCA in Aotearoa New Zealand. Demographics are similar to those previously reported in NZ, with a mean age of 74.2 years, female predominance (incidence ratio 1.59), and primarily affecting those of European descent (93%). The mean annual incidence for biopsy-proven GCA was marginally lower than that reported elsewhere in NZ (3, 4), at 10.5 per 100,000 over the age of 50, but correlates with a large population study from the UK (18), where many NZ Europeans in Canterbury are descendant. There were no differences in demographics between the LV- and C-GCA cohorts, which contrasts existing literature suggesting LV-GCA patients have a younger age at diagnosis (11, 19, 20), and may be a consequence of our small case numbers.

Clinical features at presentation were different between the two groups. C-GCA patients were more likely to present with the cardinal features of GCA such as headache and jaw claudication, while LV-GCA patients often presented with non-specific symptoms including weight loss and features of vascular insufficiency. Current literature suggests that cranial symptoms are inversely associated with LV involvement (21). Only 50% of our LV-GCA patients fulfilled the 1990 ACR classification criteria for GCA, which relies heavily upon cranial manifestations, indicating these criteria are insensitive for patients with LV disease (10, 22). Atypical presenting features and insensitive classification criteria pose challenges to the detection of disease and likely account for the diagnostic delay among patients with LV-GCA. This observation is well-described in other large cohort studies (11), and although a trend toward diagnostic delay was seen in our LV-GCA cohort, statistical significance was not met. A high index of suspicion is required to diagnose patients presenting with non-specific symptoms. In our cohort patients with LV-GCA were less likely to undergo a biopsy compared to those with limited cranial disease (*p* = 0.008); it is unclear whether the decision not to pursue biopsy was due to a low index of disease suspicion or anticipated low test yield. Of those who did undergo a biopsy, there was no difference in rates of biopsy positivity (*p* = 0.38). This contrasts current literature, which suggests LV-GCA patients are less likely to yield a positive biopsy result (11), posing further challenges to a timely diagnosis.

The extent and distribution of LV involvement can vary considerably and the reason for such a diverse spectrum of disease remains poorly understood. Our results confirm that LV-GCA has a predilection for proximal branches of the aorta (19), with highest rates of vasculitis identified in the upper limb and vertebral arteries. A relatively low number of GCA patients in our cohort had LV vasculitis detected (7%). This figure aligns with earlier GCA studies, where routine imaging of large vessels was not undertaken and LV-GCA was estimated to account for 3–15% of all GCA cases (8). More recently, prospective studies with dedicated LV imaging have reported rates of LV involvement from 29 to 83% (23, 24). Ongoing variability in reported rates is due to the broad spectrum of clinical presentations, use of various imaging modalities and inconsistent disease definitions. LV imaging of patients with GCA was not routinely conducted in Canterbury during the study period and may explain why detection rates of LV-GCA in our cohort align more closely with earlier GCA studies.

Patients with LV- and C-GCA were universally managed with corticosteroid therapy, with no difference in starting dose of oral prednisolone. Although follow-up data was limited, patients with LV-GCA were more likely to remain on steroids 5 years after diagnosis. This observation is similarly reflected in large cohort studies (25). Current literature is conflicting but suggests that LV-GCA patients may have higher relapse rates compared to C-GCA, with higher cumulative corticosteroids exposure long term (11, 26), and may explain the increased rates of steroid use at 5 years in our LV-GCA cohort. Follow-up data from our study is insufficient to comment on relapse rates, as most relapses were managed by the GP in the primary care setting. Two LV-GCA patients were diagnosed with thoracic aortic dilation, without meeting criteria for aneurysm, a rate significantly higher than that observed in C-GCA patients (*p* = 0.004). LV-GCA is a recognized risk factor for aortic complications, with potentially catastrophic complications that may not present until years after diagnosis (14–16). Routine screening for LV disease has not been adopted globally, although recent guidelines, including those published by the ACR/Vasculitis Foundation, recommend all patients with newly diagnosed GCA undergo non-invasive vascular imaging to evaluate large vessel involvement and facilitate long term surveillance of potential disease sequelae (27, 28). The resource implications of such an approach, particularly in settings with limited access to advanced imaging, are not insignificant and there remains lack of clear consensus about management and follow-up.

The main limitation of this study is its small case numbers. Only 10 patients with LV-GCA were detected, which limits the statistical power of the analysis and possibly explains the absence of observations that are consistently described in larger cohorts, such as younger age and longer delays to diagnosis for LV-GCA patients. Follow-up data was particularly limited, which is a consequence of the retrospective study design and a reflection of the health care system in NZ, where many chronic diseases are managed in the primary care setting. Another limitation of this study relates to case ascertainment. While it is expected that the majority of patients with GCA were managed in the public healthcare system, and temporal artery biopsies analyzed by Canterbury Health Laboratories, the methods applied for case identification may have missed patients managed privately, or in the primary care setting. The private sector makes up a small portion of the healthcare landscape in NZ. Access was granted to review a representative sample of private patients’ case notes, with no cases of GCA identified, suggesting few patients with GCA are seen privately. Numerous methods for case ascertainment were applied to capture such patients; however, it is not possible to quantify how many may have been missed across various sectors. While this number is expected to be small, it may explain why the incidence seen in our cohort is slightly lower than that reported by Abdul-Rahman and Nagarajah (3, 4). Finally, the cases of LV-GCA may suffer from selection bias, as imaging was performed at the discretion of the treating clinician, and therefore only those with symptoms of LV extra-cranial involvement underwent LV imaging. A prospective study with LV imaging of all consecutive GCA patients would be required to eliminate such bias.

This study is the first of its kind to describe the clinical characteristics of patients with LV-GCA in New Zealand. Incidence was comparable to previous NZ studies, and although case numbers were small, two distinct subsets of disease were apparent. Those with cranial disease were more likely to present with the cardinal clinical features of headache and jaw claudication, while patients with LV-disease often presented with non-specific symptoms including vascular insufficiency and were less likely to fulfill the ACR classification criteria for GCA. In general, treatment approach was similar, however, those with LV-GCA had greater long term steroid burden, suggesting these patients may have a more refractory disease course and require a tailored therapeutic approach. A large prospective study with LV imaging of all consecutive GCA patients is required confirm our findings, but these results suggest that an innovative diagnostic and therapeutic approach may be required to manage patients with large vessel disease.

## Data availability statement

The raw data supporting the conclusions of this article will be made available by the authors, without undue reservation.

## Ethics statement

The studies involving human participants were reviewed and approved by the University of Otago, Human Research Ethics Committee (Reference: H21/065). Written informed consent for participation was not required for this study in accordance with the national legislation and the institutional requirements.

## Author contributions

SAL, LS, SL, CH, PC, CR, and ES: contribution to study conception and design, data analysis and interpretation, drafting the article, critical revision, and final approval of the manuscript. SAL, LS, CH, PC, and CR: contribution to data acquisition. All authors contributed to the article and approved the submitted version.

## Footnotes

1
https://www.stats.govt.nz/

## References

[1] Li KJ Semenov D Turk M Pope J. A meta-analysis of the epidemiology of giant cell arteritis across time and space. *Arthritis Res Ther.* (2021) 23:82. 10.1186/s13075-021-02450-w 33706808 PMC7948334

[2] Sharma A Mohammad AJ Turesson C. Incidence and prevalence of giant cell arteritis and polymyalgia rheumatica: a systematic literature review. *Semin Arthritis Rheum.* (2020) 50:1040–8. 10.1016/j.semarthrit.2020.07.005 32911281

[3] Abdul-Rahman AM Molteno AC Bevin TH. The epidemiology of giant cell arteritis in Otago, New Zealand: a 9-year analysis. *N Z Med J.* (2011) 124:44–52. 21475359

[4] Nagarajah R Gupta R Kumar S. Diagnostic use of ultrasound in giant cell arteritis in Counties Manukau District Health Board, New Zealand. *Rheumatol Adv Pract.* (2022) 6:rkac040. 10.1093/rap/rkac040 35663155 PMC9154064

[5] De Smit E Clarke L Sanfilippo PG Merriman TR Brown MA Hill CL Geo-epidemiology of temporal artery biopsy-positive giant cell arteritis in Australia and New Zealand: Is there a seasonal influence? *RMD Open.* (2017) 3:e000531. 10.1136/rmdopen-2017-000531 29225921 PMC5706482

[6] Hutchinson J. Diseases of the arteries. *Arch Surg.* (1890) 1:323–33.

[7] Horton BM Magath TB Brown GE. An undescribed form of arteritis of the temporal vessels. *Proc Staff Meet Mayo Clinic.* (1932) 7:700–1. 17212686

[8] Klein RG Hunder GG Stanson AW Sheps SG. Large artery involvement in giant cell (temporal) arteritis. *Ann Intern Med.* (1975) 83:806–12. 10.7326/0003-4819-83-6-806 1200525

[9] Dejaco C Duftner C Buttgereit F Matteson EL Dasgupta B. The spectrum of giant cell arteritis and polymyalgia rheumatica: revisiting the concept of the disease. *Rheumatology.* (2017) 56:506–15. 10.1093/rheumatology/kew273 27481272

[10] Hunder GG Bloch DA Michel BA Stevens MB Arend WP Calabrese LH The American College of Rheumatology 1990 criteria for the classification of giant cell arteritis. *Arthr Rheum.* (1990) 33:1122–8. 10.1002/art.1780330810 2202311

[11] Muratore F Kermani TA Crowson CS Green AB Salvarani C Matteson EL Large-vessel giant cell arteritis: a cohort study. *Rheumatology.* (2015) 54:463–70. 10.1093/rheumatology/keu329 25193809 PMC4425829

[12] Evans JM O’Fallon WM Hunder GG. Increased incidence of aortic aneurysm and dissection in giant cell (temporal) arteritis: a population-based study. *Ann Intern Med.* (1995) 122:502–7. 10.7326/0003-4819-122-7-199504010-00004 7872584

[13] Nuenninghoff DM Hunder GG Christianson TJH McClelland RL Matteson EL. Mortality of large-artery complication (Aortic Aneurysm, Aortic Dissection, and/or Large-Artery Stenosis) in Patients with Giant Cell Arteritis: a population-based study over 50 years. *Arthr Rheum.* (2003) 48:3532–7. 10.1002/art.11480 14674005

[14] Bongartz T Matteson EL. Large-vessel involvement in giant cell arteritis. *Curr Opin Rheumatol.* (2006) 18:10–7. 10.1097/01.bor.0000197996.04709.4e 16344614

[15] Nuenninghoff DM Warrington KJ Matteson EL. Concomitant giant cell aortitis, thoracic aortic aneurysm, and aortic arch syndrome: occurrence in a patient and significance. *Arthr Rheum.* (2003) 49:858–61. 10.1002/art.11453 14673975

[16] Gonzalez-Gay MA Garcia-Porrua C Pineiro A Pego-Reigosa R Llorca J Hunder GG. Aortic aneurysm and dissection in patients with biopsy-proven giant cell arteritis from northwestern Spain: a population-based study. *Medicine.* (2004) 83:335–41. 10.1097/01.md.0000145366.40805.f8 15525845

[17] Koster MJ Matteson EL Warrington KJ. Large-vessel giant cell arteritis: Diagnosis, monitoring and management. *Rheumatology.* (2018) 57(Suppl. 2):ii32–42. 10.1093/rheumatology/kex424 29982778

[18] Petri H Nevitt A Sarsour K Napalkov P Collinson N. Incidence of giant cell arteritis and characteristics of patients: data-driven analysis of comorbidities. *Arthritis Care Res.* (2015) 67:390–5. 10.1002/acr.22429 25132663

[19] Prieto-González S Arguis P García-Martínez A Espígol-Frigolé G Tavera-Bahillo I Butjosa M Large vessel involvement in biopsy-proven giant cell arteritis: prospective study in 40 newly diagnosed patients using CT angiography. *Ann Rheum Dis.* (2012) 71:1170–6. 10.1136/annrheumdis-2011-200865 22267328

[20] Brack A Martinez-Taboada V Stanson A Goronzy JJ Weyand CM. Disease pattern in cranial and large-vessel giant cell arteritis. *Arthr Rheum.* (1999) 42:311–7. 10.1002/1529-0131(199902)42:2<311::AID-ANR14>3.0.CO;2-F 10025926

[21] Nuenninghoff DM Hunder GG Christianson TJ McClelland RL Matteson EL. Incidence and predictors of large-artery complication (aortic aneurysm, aortic dissection, and/or large-artery stenosis) in patients with giant cell arteritis: a population-based study over 50 years. *Arthr Rheum.* (2003) 48:3522–31. 10.1002/art.11353 14674004

[22] Seeliger B Sznajd J Robson JC Judge A Craven A Grayson PC Are the 1990 American College of Rheumatology vasculitis classification criteria still valid? *Rheumatology.* (2017) 56:1154–61. 10.1093/rheumatology/kex075 28379475 PMC6251621

[23] Blockmans D de Ceuninck L Vanderschueren S Knockaert D Mortelmans L Bobbaers H. Repetitive 18F-fluorodeoxyglucose positron emission tomography in giant cell arteritis: a prospective study of 35 patients. *Arthritis Rheum.* (2006) 55:131–7. 10.1002/art.21699 16463425

[24] Ghinoi A Pipitone N Nicolini A Boiardi L Silingardi M Germanò G Large-vessel involvement in recent-onset giant cell arteritis: a case-control colour-Doppler sonography study. *Rheumatology.* (2012) 51:730–4. 10.1093/rheumatology/ker329 22179725

[25] Sugihara T Hasegawa H Uchida H Yoshifuji H Nakaoka Y Watanabe Y Characteristics of patients with giant cell arteritis and Takayasu arteritis in a nationwide, retrospective cohort study in Japan. *Rheumatology.* (2017) 56(Supplement 3):iii52–60. 10.1093/rheumatology/kex108 31970410

[26] Sugihara T Hasegawa H Uchida HA Yoshifuji H Watanabe Y Amiya E Associated factors of poor treatment outcomes in patients with giant cell arteritis: clinical implication of large vessel lesions. *Arthritis Res Ther.* (2020) 22:72. 10.21203/rs.2.22145/v2 32264967 PMC7137303

[27] Maz M Chung SA Abril A Langford CA Gorelik M Guyatt G 2021 American College of Rheumatology/Vasculitis Foundation Guideline for the management of giant cell arteritis and takayasu arteritis. *Arthritis Rheumatol.* (2021) 73:1349–65. 10.1002/art.41774 34235884 PMC12344528

[28] Hiratzka LF Bakris GL Beckman JA Bersin RM Carr VF Casey DE Jr 2010 ACCF/AHA/AATS/ACR/ASA/SCA/SCAI/SIR/STS/SVM Guidelines for the diagnosis and management of patients with thoracic aortic disease. a report of the American College of Cardiology Foundation/American Heart Association Task Force on Practice Guidelines, American Association for Thoracic Surgery, American College of Radiology,American Stroke Association, Society of Cardiovascular Anesthesiologists, Society for Cardiovascular Angiography and Interventions, Society of Interventional Radiology, Society of Thoracic Surgeons,and Society for Vascular Medicine. *J Am Coll Cardiol.* (2010) 55:e27–129. 20359588 10.1016/j.jacc.2010.02.015

