# Supplementary material for: Giant cell arteritis: A population-based retrospective cohort study exploring incidence and clinical presentation in Canterbury, Aotearoa New Zealand
Source: Front Med (Lausanne). 2022 Nov 22;9:1057917. doi: 10.3389/fmed.2022.1057917 (PMC9723338; doi:10.3389/fmed.2022.1057917)
Supplement: Supplementary file 1 [file Table_1.DOCX]

**Supplementary File 1** – Data collection sheet
